# Costs and cost-effectiveness of Gene Xpert compared to smear microscopy for the diagnosis of pulmonary tuberculosis using real-world data from Arsi zone, Ethiopia

**DOI:** 10.1371/journal.pone.0259056

**Published:** 2021-10-25

**Authors:** Abdene Weya Kaso, Alemayehu Hailu

**Affiliations:** 1 School of Public Health, College of Medicine and Health Science, Dilla University, Dilla, Ethiopia; 2 Department of Global Public Health and Primary Care, Bergen Centre for Ethics and Priority Setting, University of Bergen, Bergen, Norway; The University of Georgia, UNITED STATES

## Abstract

**Background:**

Early diagnosis and treatment are one of the key strategies of tuberculosis control globally, and there are strong efforts in detecting and treating tuberculosis cases in Ethiopia. Smear microscopy examination has been a routine diagnostic test for pulmonary tuberculosis diagnosis in resource-constrained settings for decades. Recently, many countries, including Ethiopia, are scaling up the use of Gene Xpert without the evaluation of the cost and cost-effectiveness implications of this strategy. Therefore, this study evaluated the cost and cost-effectiveness of Gene Xpert (MTB/RIF) and smear microscopy tests to diagnosis tuberculosis patients in Ethiopia.

**Methods:**

We compared the costs and cost-effectiveness of tuberculosis diagnosis using smear microscopy and Gene Xpert among 1332 patients per intervention in the Arsi zone. We applied combinations of top-down and bottom-up costing approaches. The costs were estimated from the health providers’ perspective within one year (2017–2018). We employed “cases detected” as an effectiveness measure, and the incremental cost-effectiveness ratio was calculated by dividing the changes in cost and change in effectiveness. All costs and incremental cost-effectiveness ratio were reported in 2018 US$.

**Results:**

The unit cost per test for Gene Xpert was $12.9 whereas it is $3.1 for AFB smear microscopy testing. The cost per TB case detected was $77.9 for Gene Xpert while it was $55.8 for the smear microscopy method. The cartridge kit cost accounted for 42% of the overall Gene Xpert’s costs and the cost of the reagents and consumables accounted for 41.3% ($1.3) of the unit cost for the smear microscopy method. The ICER for the Gene Xpert strategy was $20.0 per tuberculosis case detected.

**Conclusion:**

Using Gene Xpert as a routine test instead of standard care (smear microscopy) can be potentially cost-effective. In the cost scenario analysis, the price of the cartridge, the number of tests performed per day, and the life span of the capital equipment were the drivers of the unit cost of the Gene Xpert method. Therefore, Gene Xpert can be a part of the routine TB diagnostic testing strategy in Ethiopia.

## Introduction

Tuberculosis (TB) remains a considerable public health threat in Africa [1]. It is also the leading cause of mortality in Ethiopia with a TB incidence of 163 cases per 100,000 population reported in 2016. In 2015, the prevalence of multiple drug-resistant TB (MDR-TB) reached 2.7% in new TB cases and 14.0% in retreated TB cases in Ethiopia [2,3].

Early case detection and treatment of TB cases were among the key strategies of the National TB and Leprosy Control Program (NTLCP). Thus, an advanced diagnostic tool is required to detect and treat TB in Ethiopia [4]. In Ethiopia, smear microscopy was a routine TB diagnostic tool for decades [5]. It has low sensitivity, doesn’t enable the detection of MDR-TB, and has little value in extrapulmonary TB and children [6,7]. Therefore, the detection of MDR-TB strains requires the introduction of more sensitive and highly advanced diagnostic tools. Gene Xpert is one of these advanced technologies and has the potential for rapid diagnosis of TB and MDR-TB [8,9]. In 2010, the World Health Organization (WHO) endorsed this rapid and advanced molecular tool for the diagnosis of tuberculosis [10]. According to the recent systematic review, Gene Xpert has 85% pooled sensitivity and 98% pooled specificity in TB case detection whereas it has 96% pooled sensitivity and 98% pooled specificity in Rifampicin resistance detection [11]. Furthermore, Gene Xpert has certain benefits over the routine Acid-fast bacilli (AFB) smear examination method. It has low human resource requirements and it doesn’t need biosafety during diagnosis [12]. This has generated a new hope in populations with high burdens of TB such as Ethiopia [13].

However, the scale-up of Gene Xpert as a routine diagnostic test in the health care delivery system has substantial economic implications. A previous study shows that using Gene Xpert for diagnosis of TB in routine services among suspected patients increases the health system testing costs compared with using smear microscopy [14]. Recently, in Ethiopia, Gene Xpert was used for routine TB diagnosis without evaluating the cost and cost-effectiveness implications of the method [15]. Therefore, this study aimed to evaluate the cost and cost-effectiveness of Gene Xpert (MTB/RIF) and smear microscopy tests to diagnose TB in Ethiopia.

## Methods

### Study setting

A cost and cost-effectiveness analysis of TB diagnostic strategies was conducted among 1332 patients per intervention in public health facilities in the Arsi zone. The zone comprises 28 Woreda and two town administrations. It also consists of 7 hospitals and 104 health centers. The current Ethiopian health tier system classified health facilities as a primary, secondary, and tertiary healthcare systems. In this study, one tertiary level healthcare (i.e. Regional referral hospital), one secondary level healthcare (i.e. General hospital), and six primary healthcare facilities (three hospitals and three health centers) were included. Among these facilities, one referral and general hospital perform TB testing using the Gene Xpert technique whereas the three hospitals and health centers use the AFB smear microscopy method. In the hospitals and health centers, the laboratory operates under NTLCP of Ethiopia.

### Study design and description of interventions

This study compared the cost and cost/effectiveness of two TB diagnostic methods: Gene Xpert MTB/RIF assay and smear microscopy method. A single spot sputum specimen was tested for all suspected TB cases in the Gene Xpert methods (MTB/RIF assay). A fresh sputum sample was taken from the patients presented with signs and symptoms of pulmonary TB based on WHO TB and Ethiopia NTLCP screening criteria [10,16]. The sputum sample was combined with the reagent in a 2:1 ratio for sputum liquefaction and inactivation. Then, it was incubated at room temperature for 15–20 minutes. After incubation, a total of 2 ml of the mixture was introduced into the Gene Xpert cartridge and loaded into the Gene Xpert instrument for analysis. The instrument generates a test report automatically within 2 hours. In the smear microscopy method, two spot-spot sputum samples (i.e. a first spot sputum sample at the first arrival of patients to laboratory and another one spot sample after 30 minute) were collected from all suspected TB patients and examined for acid-fast bacilli using Ziehl–Nielsen or fluorescent staining technique [13,17].

### Data collection

The cost estimation was based on service data collected from three health centers and five hospitals in the Arsi Zone. This costing was conducted from the health providers’ perspective, with all costs to the health system for diagnosing TB among suspected individuals with smear microscopy and the Gene Xpert technique were included. Various cost components, such as building space, equipment, consumables, overhead, and human resources (HR), were included. Additionally, weekly quality control, external quality control, and annual calibration costs for Gene Xpert were included. However, the maintenance cost for both methods was not included due to a lack of data.

The cost data were collected from hospital procurement invoices, expert interviews, managers’ opinions, and other administrative reports available in the health facilities. The person-time elapsed per test was estimated based on observation and consultation with laboratory technicians. The cost-of-training data were obtained from Oromia Public Health Research Capacity Building & Quality Assurance Laboratory. The annual calibration cost was obtained from the Stop TB Partnership source [18]. The cost types and quantity of each resource used in each diagnostic technique were recorded using a Microsoft Excel 2010 spreadsheet. The data collection tools were developed by modifying the WHO guidelines for the cost and cost-effectiveness analysis of TB control. After the pretest was conducted, necessary corrections were made to the data collection tools.

### Data analysis

The cost estimation was conducted with a one-year time frame (2017–2018) using an ingredients-based and top-down approach. In ingredient-based approach, all inputs required to perform diagnostic testing was identified, quantified and multiplied with their prices to arrive at a unit cost per patient tested. Moreover, in top-down method, we divided the gross cost data expenditure on building, overhead, reagents and equipment allocated specifically to each diagnostic method by the numbers of tests conducted. We valued personnel cost based on an estimated proportion of working time spent on TB diagnosis by each method. The smear microscopy diagnostic procedure took 35 minutes per test, and the Gene Xpert method took 25 minutes per test. The number of training days for conducting tests by AFB smear microscopy and Gene Xpert was assumed to be five days for smear microscopy and three days for Gene Xpert technique. The cost of reagents and consumables for smear microscopy was calculated using the gross cost of each reagent over the number of patients tested with that amount. Moreover, the cost of the Xpert cartridge kit was obtained from a published source [19].

The cost of equipment for each diagnostic method was obtained by dividing the equipment’s annualized cost by the number of tests performed. The useful life of microscopy was assumed to be 10 years, running five tests per day. A Gene Xpert machine was assumed to be operating four simultaneous tests and running for eight hours per day. The instrument was assumed to have a useful life of 10 years and to process, on average, eight sputum samples per day. For buildings, an expected lifetime of 30 years was used. Laboratory space cost was allocated based on the proportional size required for conducting TB diagnosis.

The cost of quality control for smear microscopy was estimated by identifying all the resources needed to perform the quality control procedure based on WHO Stop TB guidelines. The cost of overhead for each technique was calculated by taking 5% of the total health overhead cost based on laboratory heads’ expert opinions. Capital costs were annualized using a 3% discount rate per year [20,21]. Local costs were collected in Ethiopian birr and converted to United States dollars (US$) using average exchange rates from the National Bank of Ethiopia (US$1 = 27.18 birr) [22]. All the costs were adjusted for inflation using the consumer price index of the year 2018 as a base year cost.

An incremental cost-effectiveness ratio (ICER) was calculated using the ratio of the change in unit cost per case detected. As there is no widely accepted willingness-to-pay (WTP) threshold for this intermediate outcome (i.e., ICER in terms of cost per case detected), we did not use any threshold, and therefore we presented only the estimated ICER in this study.

### Ethics approval and consent to participate

Ethical approval was obtained from the Addis Ababa University School of Public Health Institutional Review Board. The ethical review board provided a waiver for consent to participate, as the data were collected from patient records.

## Results

### Socio-demographic characteristics of the participants

Among TB suspected patients diagnosed by AFB smear microscopy method, 692 (52%) were male, and 639(48%) were in the age group 5–25 years old. Around 1,258 (94.4%) patients had a smear-negative result, whereas 74 (5.6%) had a smear-positive result. Among 1332 patients enrolled in the Gene Xpert algorithm, more than half (54.4%) were males and around 221 (16.6%) patients have TB infection whereas the remaining 1111(83.4%) were negative for TB. The majority of patients diagnosed using the Gene Xpert method were in the age group 5–25 years with the mean age of 34± 17 standard deviation (SD) (Table 1).

**Table 1:** Characteristics of TB suspected patients by testing technique.

Category	Smear microscopy Frequency (%)	Gene Xpert Frequency (%)
**Age**		
5–25 years	639(48%)	504(37.8%)
26–34 years	348(26.1%)	279(21%)
35–44 years	174(13.1%)	199(14.9%)
45–54 years	124(9.3%)	144(10.8%)
55 and above years	47(3.5%)	206(15.5%)
**Sex**		
Male	692 (52.0%)	724 (54.4%)
Female	640 (48.0%)	608 (45.6%)
**Test results**		
Test positive	74 (5.6%)	221 (16.6%)
Test negative	1,258 (94.4%)	1,111(83.4%)

### Cost per patient tested

The unit cost of testing suspected TB patients using smear microscopy and the Gene Xpert algorithm varies with the volume of testing and level of health facilities. The average cost of the testing using the smear microscopy technique was $3.1 (ranging from $2.40 to $4.96) whereas it was $12.9 for the Gene Xpert method (ranging from $12.69 to $13.22). Consumables accounted for 41.3% of AFB smear examination costs while the Xpert cartridge cost was the major determinants of Gene Xpert’s unit cost. The staff salary costs accounted for 29% of the Gene Xpert technique whereas it was 5.4% ($0.9 per test) for AFB smear microscopy (Table 2).

**Table 2:** Unit cost per patient tested of smear microscopy and the Gene Xpert diagnostic method (2018 US$).

Health facilities	Consumable	Overhead and space	Equipment	HR	Tested (annual)	Unit cost
**Smear microscopy**	**1.3**	**0.4**	**0.7**	**0.9**		**3.1**
Bokoji HSP	375.1	106.7	210.7	265.5	343	2.8
Bokoji HC	241.0	59.3	64.6	190.3	167	3.3
Kersa HSP	214.1	103.3	151.9	170.2	129	5.0
Sagure HC	312.3	59.4	67.9	189.9	262	2.4
Gobesa HSP	272.0	103.3	188.0	192.3	209	3.6
Meraro HC	285.6	58.2	66.8	183.6	222	2.7
**Gene Xpert**	**10.7**	**0.2**	**1.3**	**0.7**		**12.9**
Asella HSP	8,003.0	130.2	882.2	543.4	753	12.7
Didea HSP	6,204.2	102.1	879.1	466.1	579	13.2

**Note**: HR = human resources; HC = health center; HSP = hospital.

### Cost per TB case detected

In AFB smear microscopy techniques, the unit cost per TB case detected was $55.8. The TB staining supplies accounted for 41.1% of the AFB smear examination costs whereas around $16.6 (29.7%) was attributable to overhead and equipment costs. Besides, the cost per TB case detected for the Gene Xpert testing technique was $77.9. The medical supplies (i.e., cartridge kit) cost accounted for 82.5% of the Gene Xpert costs. The ICER for the Gene Xpert techniques compared to the AFB smear microscopy method was $20.0 per TB case detected (Tables 3 and 4).

**Table 3:** Cost per case detected of smear microscopy and Gene Xpert diagnostic methods by health facilities in Arsi Zone, Ethiopia, 2019 (2018 US$).

Health facilities	Supplies	Overhead and space	Equipment	HR	Number of cases detected	Cost per case detected
***Smear microscopy***	**23.0**	**6.6**	**10.0**	**16.1**	**74**	**55.8**
Bokoji HSP	18.7	5.3	10.5	13.3	20	47.9
Bokoji HC	24.1	5.9	6.5	19.0	10	55.5
Kersa HSP	21.4	10.3	15.2	17.0	10	63.9
Sagure HC	26.0	4.9	5.6	15.8	12	52.5
Gobesa HSP	17.0	6.4	11.7	12.0	16	47.2
Meraro HC	47.6	9.7	11.1	30.6	6	99.0
***GeneXpert***	**64.3**	**1.0**	**8.0**	**4.6**	**221**	**77.9**
Asella HSP	68.4	1.1	7.5	4.6	117	81.7
Didea HSP	59.7	1.0	8.4	4.5	104	73.6

**Note**: HR = human resources; HC = health center; HSP = hospital.

**Table 4:** Incremental cost-effectiveness ratios of GeneXpert compared to smear microscopy.

Diagnostic methods	Cost per case detected	Incremental cost per case detected
Smear microscopy	55.8	Ref.
GeneXpert	77.8	20.0

Ref: Reference strategy (smear microscopy was the reference strategy). Effectiveness was measured in terms of TB cases detected.

### Scenario analysis of the costs

The reduction of the capital equipment’s shelf life time from 10 to five years drives the unit cost of AFB smear examination to $3.60. Likewise, the unit cost would reduce by 16.13% as the volume of tests performed increased from 5 to 10 per day. Besides this, the full utilization of microscopy used to the AFB smear examination at health centers would increase the test’s cost by 7%.

In the Gene Xpert technique, the reduction of the cartridge price by 10% reduces the test’s cost to $11.7. In addition, the increment of the number of tests executed per day from eight to 16 reduces the test’s costs by 8.1%. However, the cost per test would increase to $13.8 if the life span of the four-module Gene Xpert machine reduced from ten to five years (Table 5).

**Table 5:** Scenario analysis of cost assumptions for smear microscopy and Gene Xpert diagnostic methods (2018US$).

Parameter	Cost per test	Change (%)	Cost per case detected	Change (%)
***Smear microscopy***				
**Base-case unit cost**	**3.1**		**55.8**	
Reduce useful life of capital equipment from 10 to 5 years	3.6	+15.8	64.6	+15.7
Increase the number of tests to 10 per day	2.6	-16.1	46.7	-16.4
Allocate 100% of microscopy to smear microscopy at the health center	3.3	+7.1	59.7	+6.9
***GeneXpert***				
**Base-case unit cost**	**12.9**		**77.9**	
Reduce the useful life of capital equipment from 10 to 5 years	13.8	+7.0	83.4	+7.1
Increase the number of tests to 16 per day	11.9	-8.1	71.6	-8.1
Reduce the price of the cartridge by 10%	11.7	-9.8	70.3	-9.7

## Discussion

Tuberculosis is a global public health threat especially in sub-Saharan African countries [1]. The introduction of Gene Xpert technology has offered the potential to increases the case detection rate of TB and MDR-TB in the health service delivery of low and middle-income countries [23]. This study evaluated the cost and cost-effectiveness of Gene Xpert and AFB smear microscopy in TB diagnosis. In our study, the use of the Gene Xpert algorithm is a cost-effective intervention from the health provider’s perspective with an ACER of $78. This is consistent with a recent systematic review which suggests that using Gene Xpert in a routine health care delivery system is cost-effective in various settings [11]. Our findings are also in line with a study from South African that indicated using Gene Xpert for routine TB diagnosis was a cost-saving method [24].

In this study, the cost per test for Gene Xpert diagnostic method ($12.9) was substantially lower than AFB smear microscopy techniques ($3.1). In our cost-scenario analysis, the volume of tests is an essential factor influencing the unit cost of the diagnostic methods. As the number of tests per day increases, the unit cost per test would decrease for both smear microscopy and Gene Xpert. Our finding is consistent with studies from Sub-Saharan African countries that reported a similar trend, and the primary explanation for the discrepancy in unit cost across health facilities by test volume was that facilities incurred the fixed costs (i.e., equipment, HR, etc.) regardless of the number of tests performed [4,25–29].

Even though we found that Gene Xpert is a cost-effective diagnostic method, its unit cost per test is higher than the AFB smear examination technique. The relatively high cost of Gene Xpert is attributable to the high prices of the equipment and cartridge. Moreover, in our study, the highest share of the unit cost of the Gene Xpert method is attributable to supplies (42%) especially cartridge costs. This estimate was in line with reports from South African and Ugandan studies’ which indicated that the cartridge’s cost accounted for the largest share of the Gene Xpert technique’s unit cost [20,30]. Other studies’ findings from settings in low- and middle-income countries consistently reported the same trends that Xpert cartridge costs are critical drivers of the overall cost [31–33]. Thus, the high cost of cartridges can be a barrier in the scale-up of Gene Xpert as a routine TB testing intervention. Therefore, to ensure this technology’s financial sustainability in low-income settings, reducing the cartridge and equipment price is essential [34].

To the best of our knowledge, this is the first study that evaluated the cost and cost-effectiveness of Gene Xpert compared to smear microscopy in Ethiopian settings. However, this study has few drawbacks. First, the maintenance cost for the diagnostic machines was not included in this analysis. Excluding the cost of maintenance might significantly affect the total unit cost of the diagnostic methods [35,36]. Furthermore, although the information on the diagnosis accuracy of the techniques in TB-HIV co-infection cases would be relevant, because of the data limitation, TB-HIV co-infection was not included. Additionally, we used an intermediate outcome in this study (i.e., the ICER is presented in cost per TB case detected). Thus, the use of 1-times or 3-times gross domestic product per capita per DALY averted as a WTP threshold to determine the strategies’ cost-effectiveness may not be directly applicable to our study.

## Conclusion

In conclusion, using the Gene Xpert diagnostic method in routine TB management compared to smear microscopy was cost-effective. In the cost scenario analysis, the price of the cartridge, the number of tests performed per day, and the life span of the capital equipment were the drivers of the unit cost of Gene Xpert techniques. Therefore, Gene Xpert can be considered as a part of the routine TB diagnostic testing strategy in Ethiopia.

## Supporting information

S1 Dataset (RAR)

## Abbreviations

AFB: Acid Fast Bacilli
ACER: average cost-effectiveness ratio
DALY: Disability-Adjusted Life Year
HR: human resources
HIV: human immunodeficiency virus
ICER: incremental cost-effectiveness ratio
MDR-TB: multi-drug resistant TB
MDR/RIF: multi-drug resistant TB/rifampicin
MTB: mycobacterium TB
TB/HIV: TB/human immune virus
PSA: probabilistic sensitivity analysis
TB: tuberculosis
US$: United States dollar
WTP: Willingness-to-pay
WHO: World Health Organization
